# Psoriasis and Its Impact on In-Hospital Outcome in Patients Hospitalized with Acute Kidney Injury

**DOI:** 10.3390/jcm9093004

**Published:** 2020-09-17

**Authors:** Johannes Wild, Lukas Hobohm, Thomas Münzel, Philip Wenzel, Kerstin Steinbrink, Susanne Karbach, Karsten Keller

**Affiliations:** 1 Center for Thrombosis and Hemostasis (CTH), University Medical Center Mainz (Johannes Gutenberg-University Mainz), 55131 Mainz, Germany; Johannes.Wild@unimedizin-mainz.de (J.W.); wenzelp@uni-mainz.de (P.W.); susannekarbach@gmx.de (S.K.); karsten.keller@unimedizin-mainz.de (K.K.); 2 Department of Cardiology, University Medical Center Mainz (Johannes Gutenberg-University Mainz), 55131 Mainz, Germany; tmuenzel@uni-mainz.de; 3 Department of Dermatology, University Medical Center Münster, Westfälische Wilhelms-University Münster, 48149 Münster, Germany; Kerstin.Steinbrink@ukmuenster.de; 4 Department of Sports Medicine, Medical Clinic VII, University Hospital Heidelberg, 69120 Heidelberg, Germany

**Keywords:** AKI, psoriasis, mortality, trends, dermatoepidemiology

## Abstract

Background: Psoriasis is a chronic inflammatory disease which affects the body far beyond the skin. Whereas there is solid evidence that chronic skin inflammation in psoriasis drives cardiovascular disease, the impact on renal impairment and acute kidney injury (AKI) is still unclear. We aimed to analyze the impact of psoriasis on the in-hospital outcome of patients hospitalized with AKI. Methods: In this retrospective database study, we investigated data on characteristics, comorbidities, and in-hospital outcomes for all hospitalized patients with AKI stratified for concomitant psoriasis, which were collected by the Federal Office of Statistics in Germany between 2005 and 2016. Results: Among the 3,162,449 patients treated for AKI in German hospitals between 2005 and 2016, 11,985 patients (0.4%) additionally suffered from psoriasis. While the annual number of AKI patients with psoriasis increased significantly from 485 cases (4.0%) in 2005 to 1902 (15.9%) in 2016 (*p* < 0.001), the in-hospital mortality decreased substantially (from 24.9% in 2005 to 17.4% in 2016; *p* < 0.001). AKI patients with concomitant psoriasis were younger (70 (IQR; 60–78) vs. 76 (67–83) years; *p* < 0.001) and were more often treated with dialysis (16.3% vs. 13.6%, *p* < 0.001). Presence of psoriasis in AKI patients was associated with reduced prevalence of myocardial infarction (OR 0.62; *p* < 0.001), stroke (OR 0.85; *p* = 0.013), and in-hospital mortality (OR 0.75; *p* < 0.001). Conclusions: AKI patients with psoriasis were hospitalized in median 6 years earlier than those without. Despite younger age, we detected higher use of kidney replacement therapy in patients with psoriasis, indicating a more severe course of AKI. Our findings might improve management of these patients and contribute evidence for extracutaneous, systemic manifestations of psoriasis.

## 1. Introduction

For many years, the medical community classified psoriasis as an auto-immune disease which exclusively affects the skin [1]. With a reported prevalence ranging between 2% and 6% in industrialized nations [2], the number of patients suffering from the disease is of remarkable prevalence. Over the last decades, a growing body of evidence has revealed that psoriasis is a chronic multi-systemic disease that is associated with a multitude of comorbidities and psoriatic patients die about three to six years earlier than non-affected individuals [3,4,5,6,7].

Psoriasis appeared to be an important and life-threatening risk factor particularly when leading to cardiovascular as well as metabolic comorbidities and their acute manifestations (for example, myocardial infarction or sudden cardiac death) [8,9,10,11,12,13]. Patients with severe psoriasis have a higher prevalence of the classical cardiovascular risk factors such as diabetes mellitus, arterial hypertension, smoking, and obesity [10,14,15]. In addition, a higher risk for development of cardiovascular diseases independent of these traditional cardiovascular risk factors as well as an additional key driver for mortality [11]. In this context, systemic inflammation in psoriasis connecting skin and vascular disease might be of crucial relevance [16].

In contrast to the growing data regarding the link between cardiovascular diseases and psoriasis, a direct association between psoriasis and kidney diseases has been reported and debated over a long time [17], but evidence is still lacking and decryption of the patho-mechanisms remains pending [18,19]. Previous epidemiological studies in psoriasis patients showed a significantly increased risk of death associated with kidney diseases in general [20]. Nevertheless, a cause-and-effect relationship between the skin-disease psoriasis and acute kidney injury (AKI) or chronic kidney disease (CKD) has still not been established and the extent regarding the impact of psoriasis on the outcomes in the different stages of kidney disease has not been determined.

AKI is a frequent and often life-threatening condition, with a high impact on patients and healthcare systems [21]. As AKI in general is accompanied by high mortality and increases the susceptibility to chronic and end-stage kidney disease [22], we analyzed characteristics, comorbidities, and in-hospital events in hospitalized patients with AKI and concomitant psoriasis compared to those without psoriasis.

## 2. Patients and Methods

### 2.1. Data Source

The computed study analyses were performed on our behalf by the Research Data Center (RDC) of the Federal Statistical Office and the Statistical Offices of the federal states in Wiesbaden, Germany (source: RDC of the Federal Statistical Office and the Statistical Offices of the federal states, DRG Statistics 2005–2016, own calculations). The RDC provided us the data as aggregated statistical results on the basis of SPSS codes (SPSS^®^ software, version 20.0, SPSS Inc., Chicago, IL, USA), which were previously sent by us to the RDC [23,24].

### 2.2. Diagnoses, Procedural and Diagnostic Codes, and Definitions

In Germany, the diagnoses (German Diagnosis Related Groups (G-DRG) are coded according to the International Classification of Diseases and Related Health Problems, 10th Revision with German Modification (ICD−10-GM), and diagnostic, surgical, as well as interventional procedures according to the German Procedure Classification (OPS, surgery and procedures codes (Operationen- und Prozedurenschlüssel)). All DRG diagnoses and OPS codes of the hospitalized patients are gathered by the Federal Statistical Office of Germany. For the present post-hoc analysis, we identified all hospitalized patients with AKI by the ICD-code N17 between the years 2005 and 2016. Additionally, we stratified the hospitalizations with AKI for the presence of psoriasis (ICD-code L40).

The ‘Clinical Practice Guideline for Acute Kidney Injury (AKI)’ defines AKI as an increase of serum creatinine of more than 0.3 mg/dL within 48 h or an increase of more than 1.5 times baseline (which is known or presumed to have occurred within the prior 7 days) or the decrease of urine volume to less than 0.5 mL/kg/h for 6 h. This simple definition allows to diagnose AKI without using any estimating equation for glomerular filtration rate, which all have limitations in daily practice [25,26].

### 2.3. Coded Parameters and Study Outcomes

The primary endpoint of this study was defined as death of any cause during the hospital stay (all-cause in-hospital death). The second endpoint focus on an aggravated cardiovascular profile, including I) serious cardiovascular events during hospitalization such as ischemic stroke (ICD-code I63), myocardial infarction (ICD-codes I21 and I22), and pulmonary embolism (ICD-code I26), II) cardiovascular risk factors such as diabetes mellitus (ICD-codes E10-E14), essential arterial hypertension (ICD-code I10), and obesity (ICD code E66), as well as III) cardiovascular comorbidities like coronary artery disease (ICD code I25), heart failure [I50], and atrial fibrillation/flutter (ICD code I48). In addition, necessity for transfusion of erythrocyte concentrates (OPS code 8–800) as well as clinically relevant bleeding events (such as intracerebral bleeding (ICD-code I61) or gastrointestinal bleeding (ICD-code IK920, K921, and K922)) were detected and analyzed.

### 2.4. Ethical Aspects

Since this study did not involve direct access to data of individual patients by the investigators, approval by an ethics committee and informed consent was not required, in accordance with German law.

### 2.5. Statistical Methods

While continuous variables are presented as median and interquartile range (IQR), categorical variables were reported as absolute numbers and corresponding percentages. We compared inpatients suffering from AKI with and without psoriasis as well as survivors vs. non-survivors in AKI patients with psoriasis using the Mann–Whitney U test for continuous variables and the Fisher’s exact or chi-square test, as appropriate, for categorical variables. Total numbers, proportion of AKI patients with psoriasis, incidence, and relative mortality rate for patients with AKI and psoriasis were calculated annually and linear regressions were used to assess trends over time. The results were presented as beta (*β*) and corresponding 95% confidence intervals (CI).

Univariate and multivariate logistic regression models were performed to investigate the impact of age, comorbidities, and clinical conditions on the mortality during hospitalization (in-hospital mortality). Results were presented as odds ratios (OR) and corresponding 95% CIs. Multivariate logistic regression models included the following parameters for adjustment: age, sex, cancer (ICD codes C00-C97), coronary artery disease (ICD code I25), heart failure (ICD code I50), chronic obstructive pulmonary disease (COPD, ICD code J44), essential arterial hypertension, diabetes mellitus, and chronic kidney disease (CKD, comprised diagnosis of chronic kidney disease stages 3 to 5 with glomerular filtration rate <60 mL/min/1.73 m^2^, ICD-code N18.3, N18.4, N18.5, N18.83, N18.84, N18.9) in order to demonstrate statistically independence of the aforementioned parameters.

The software SPSS (SPSS^®^ software, version 20.0, SPSS Inc., Chicago, IL, USA) was used for statistical analysis. *p* values of <0.05 (two-sided) were considered to be statistically significant.

## 3. Results

### 3.1. Baseline Characteristics

The German nationwide inpatient sample comprised 3,162,449 hospitalizations of patients with AKI between 2005 and 2016. The majority of patients was male (1,712,634; 54.2%) with a median age of 76 (IQR 60–78) years. Cardiovascular comorbidities were common in these patients: Overall, 813,696 (25.7%) were diagnosed with coronary artery disease, 1,158,990 (36.6%) with heart failure and 985,758 (31.2%) with arterial fibrillation/flutter. In addition, 1,425,566 (45.1%) of the patients had a diagnosis of arterial hypertension and 1,112,335 (35.2%) were coded with diabetes mellitus (Table 1). Of those overall patients, 11,985 (0.4%) hospitalizations were coded with both AKI and psoriasis. AKI patients with concomitant psoriasis were six years younger than AKI patients without psoriasis (70 (IQR, 60–78) vs. 76 (67–83) years, *p* < 0.001) and had more frequently cardiovascular risk factors including essential arterial hypertension (50.9% vs. 45.1%, *p* < 0.001), diabetes mellitus (42.5% vs. 35.1%, *p* < 0.001), and obesity (19.3% vs. 8.8%, *p* < 0.001).

### 3.2. Impact of Psoriasis on In-Hospital Mortality in Patients Hospitalized for AKI

Serious adverse events such as myocardial infarction (4.7% vs. 6.5%, *p* < 0.001) and stroke (2.0% vs. 2.2%, *p* = 0.057) occurred less often in hospitalizations of patients with AKI and psoriasis than in those of patients without psoriasis. Importantly, patients with AKI and psoriasis died less often during the in-hospital stay compared to patients without psoriasis (22.1% vs. 28.9%, *p* < 0.001) (Table 1). The multivariate regression models confirmed that presence of psoriasis in patients with AKI was associated with a lower risk for in-hospital death (OR 0.75 (95% CI 0.72–0.79), *p* < 0.001) as well as myocardial infarction (OR 0.62 (95% CI 0.57–0.68), *p* < 0.001) and stroke (OR 0.85 (95% CI 0.75–0.87), *p* = 0.013) in the hospitalizations (Table 2). However, presence of psoriasis was independently associated with deep vein thrombosis or thrombophlebitis (OR 1.29 (95% CI 1.14–1.45), *p* < 0.001) and the need of transfusion of erythrocyte concentrates (OR 1.07 (95% CI 1.03–1.20), *p* = 0.001) in patients with AKI (Table 2).

### 3.3. Renal Replacement Therapy and Type of Procedure

In all patients hospitalized with AKI, 430,856 (13.6%) were treated with dialysis (Table 1). In AKI patients with psoriasis, the rate of dialysis was significantly higher than in patients without psoriasis (16.3% vs. 13.6%, *p* < 0.001). All kinds of renal replacement therapy were more prevalent in patients with concomitant psoriasis: hemofiltration (3.8% vs. 3.2%, *p* < 0.001), hemodialysis (14.1% vs. 11.6%, *p* < 0.001), and hemodiafiltration (4.6% vs. 3.9%, *p* < 0.001) (Table 1). The different results of the multivariate regression model (adjusted for age, gender, and comorbidities) suggest that higher renal replacement therapy rates in AKI patients with psoriasis might be most probably driven by the younger age. Furthermore, it could be affected by higher rates of CKD in stages 2, 3, and 5 in comparison to the patients with AKI without psoriasis (Table 1).

### 3.4. Trends between 2005 and 2016

**Figure 1:**
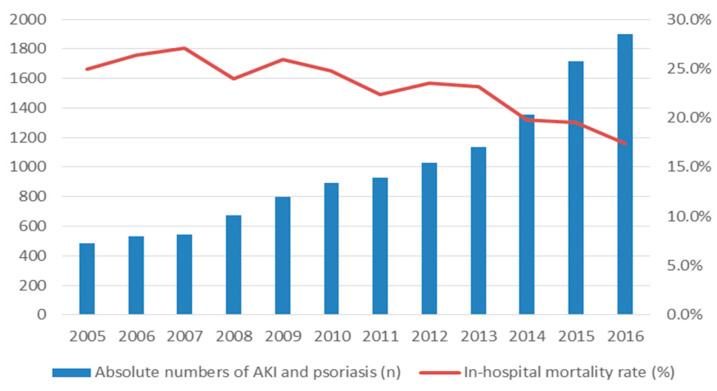
Patient’s characteristics of non-survivors versus survivors in patients with acute kidney injury (AKI) and psoriasis.

Overall, 3,162,449 patients with AKI were treated in-hospital during the observational period. Of those, the minority (0.4%) had an additionally diagnosis of psoriasis. Annual numbers of hospitalizations of AKI patients with psoriasis increased significantly from 485 cases in 2005 to 1902 cases in 2016 (*β* 0.005 (95% CI −0.05 to 0.06); *p* < 0.001), whereas the in-hospital mortality rate decreased during the same period from 24.9% in 2005 to 17.4% in 2016 (*β* −1.85 (95% CI −0.68 to −0.39); *p* < 0.001) (Figure 1). Focusing on trends of patients with AKI and psoriasis in the 11-year observational timeframe, no trend in gender distribution was observed (*β* 0.07 (95% CI −0.05 to 0.19), *p* = 0.247) (Figure S1). In contrast, comorbidities as coronary artery disease (*β* 0.21 (95% CI 0.08 to 0.35), *p* = 0.002), heart failure (*β* 0.50 (95% CI 0.38 to 0.63), *p* < 0.001), atrial fibrillation/flutter (*β* 0.40 (95% CI 0.27 to 0.53), *p* < 0.001), COPD (*β* 0.21 (95% CI 0.05 to 0.36), *p* = 0.008) increased over time. Serious adverse events such as myocardial infarction (*β* −0.12 (95% CI −0.39 to 0.16), *p* = 0.410), stroke (*β* −0.09 (95% CI −0.52 to 0.33), *p* = 0.663), and pulmonary embolism (*β* −0.29 (95% CI −0.76 to 0.18), *p* = 0.224) did not changed during the investigated period. Over the years, dialysis was less often performed (*β* −0.72 (95% CI −0.88 to −0.56), *p* < 0.001) during hospitalizations of patients with AKI and psoriasis.

Non-survivors were slightly older than survivors (71 (IQR, 61–78) vs. 70 (60–78) years, *p* < 0.001), had more frequently comorbidities as malignancy (18.6% vs. 10.4%, *p* < 0.001), heart failure (43.1% vs. 36.1%, *p* < 0.001), and arterial fibrillation/flutter (35.2% vs. 29.2%, *p* < 0.001), but considerably less often arterial hypertension (44.7% vs. 52.7%, *p* < 0.001). No differences were observed for other comorbidities. In non-survivors, bleeding events such as gastro-intestinal bleeding (6.4% vs. 3.7%, *p* < 0.001) and intracranial bleeding (1.3% vs. 0.5%, *p* < 0.001) with consecutively more frequent need of transfusion of erythrocytes concentrates (54.1% vs. 29.4%, *p* < 0.001) were observed (Table S1).

## 4. Discussion

The key findings of our study can be summarized as follows: (I) Annual numbers of AKI patients with psoriasis increased from 2005 to 2016. (II) While the in-hospital mortality decreased, patients’ characteristics shifted towards older age and more comorbidities. (III) Since hospitalizations for AKI in patients with psoriasis occurred in median 6 years earlier in life than in those AKI patients without psoriasis, (IV) patients without psoriasis were associated with a higher risk of in-hospital mortality.

The aim of our study was to contribute to the growing evidence and understanding of psoriasis as a disease with systemic effects on the cardiovascular system and beyond. Unfortunately, many physicians still consider psoriasis primarily as a cosmetic skin-deep problem. A study in Portuguese general practitioners (GP) revealed tremendous differences regarding the awareness for systemic comorbidities and elevated mortality of chronic inflammatory diseases. Compared to systemic lupus erythematoides and rheumatoid arthritis, patients with psoriasis are less often screened for arterial hypertension, diabetes mellitus, or dyslipidemia as major cardiovascular risk factors, indicating that the systemic dimension of the skin disease is often under-estimated, unrecognized, and unnoticed [27]. These findings are in line with data from the US, where most GPs and cardiologists are still not aware of the systemic character of psoriasis [28]. Furthermore, known and maybe still unknown disease-modifying factors in the daily life of those patients provide a further challenge for clinicians and patients [29,30,31,32].

Regarding the baseline characteristics, our data are in line with previous publications, which associated psoriasis with the classical cardiovascular risk factors diabetes mellitus, arterial hypertension, and obesity [15,33,34]. Remarkably, psoriasis is accompanied by a substantially aggravated cardiovascular profile of the AKI patients despite younger age. Although our cohort of patients with AKI does not differ from previous studies in patients without AKI, the approximately 7% higher prevalence of diabetes mellitus in AKI patients with psoriasis in comparison to those without psoriasis requires special attention. The development of diabetes mellitus as important cardiovascular risk factors is increased and accelerated in patients with psoriasis and diabetes mellitus [35,36] for its part enhances the risk to develop AKI and CKD [37,38]. In addition, also AKI and CKD are accompanied by higher atherosclerosis in different vascular systems, as we also found a higher prevalence of coronary artery disease in accordance with the literature [39,40,41,42,43]. Thus, (I) AKI, (II) psoriasis, (III) CKD, IV) cardiovascular as well as atherosclerotic diseases, and (V) especially, diabetes mellitus might influence each other, are linked and may trigger and boost development and aggravation of each other. However, in contrast to most previous studies [8,9,10,11,12], our findings demonstrated a lower rate of myocardial infarction, stroke, as well as in-hospital mortality in the patients with both AKI and psoriasis compared to AKI patients without psoriasis. The following suggestions might explain this surprising result:

Firstly, the substantially lower age in the group of AKI patients with psoriasis may be one of the main causes regarding lower frequency of the acute cardiovascular events and the in-hospital mortality (in median 6 years younger), demonstrated in the univariate regression model. Although the multivariate logistic regression model adjusted for age, sex, and important comorbidities showed an independent association of psoriasis with lower rates regarding stroke, myocardial infarction as well as in-hospital mortality, it has to be considered that the model cannot compensate all age-dependent effects in comorbidities, which were not included in the adjustment.

Secondly, the multivariate regression model indicates that psoriasis is singularly not an important independent risk factor for cardiovascular events and in-hospital mortality in AKI patients (since AKI on itself is a very potent driver of mortality) and the increased mortality and risk for cardiovascular events in psoriatic AKI patients might be mainly mediated by cardiovascular risk factors and cardiovascular comorbidities. After adjusting for these important cardiovascular risk factors and cardiovascular comorbidities in our multivariate regression model, psoriasis was not an independent risk factor for cardiovascular events and in-hospital mortality in this special population. Since the univariate regression models were influenced by the large age-difference between both groups, it is difficult to interpret the results of the univariate regressions.

Thirdly, we cannot exclude that the reported findings might be due to under-coding/under-reporting of comorbidities (including psoriasis) in severe AKI cases; for instance, those patients, who died before or immediately after admission, might not be coded with psoriasis. This may lead to a bias towards better in-hospital survival of AKI patients with psoriasis in comparison to those without.

Since our study results should be regarded as hypothesis generating, large prospective observational studies are needed to investigate the impact of psoriasis on renal impairment in more detail. However, our study data substantially contribute to get new insights in the triad of psoriasis, cardiovascular diseases, and AKI.

Another point of interest in our study was the prevalence of deep vein thrombosis or thrombophlebitis (DVT) in patients with AKI and concomitant psoriasis. To our best knowledge, this is the first large study focusing on this special population. In previous studies, psoriasis was reported to be a risk factor for venous thromboembolism (VTE) in patients with non-affected kidney function [44,45]. Our data are in line with these publications as we also found a higher rate of DVT in AKI patients with psoriasis. It has to be hypothesized that psoriasis with its specific vascular inflammation contributes to increased occurrence of cardiovascular events including VTE [46,47,48,49]. Interestingly, the higher rate of DVT did not result in a higher rate of pulmonary embolism in our cohort of AKI patients with psoriasis.

The baseline characteristics of our study confirm the higher prevalence of CKD in psoriasis. Whereas previous studies could already show a higher prevalence of CKD in psoriasis in general [19,50,51,52], we now can contribute data of pre-existing CKD in the cohort of AKI patients with psoriasis. We demonstrate that CKD stages 2, 3, and 5 were more common in our cohort of AKI patients with psoriasis—despite the significantly younger age of the patients. Thus, CKD seems relevant in psoriasis and affecting the patients at a significantly lower age. We interpret these findings in close connection to the higher prevalence of other comorbidities, especially arterial hypertension and diabetes mellitus. Regarding renal replacement therapies, patients with AKI and psoriasis were more often treated with dialysis. After multivariate adjustment, we observed that this trend might most probably be depending on the younger age and influenced by CKD of the patients in this cohort.

From 2005 to 2016, the annual number of AKI patients with psoriasis increased, whereas the in-hospital mortality decreased significantly. During these years, biologics targeting tumor necrosis factor-alpha (TNF-α), interleukin (IL)−12/23 and IL−17 started to provide new and efficient treatment options as a second-step therapy for patients with moderate to severe psoriasis or for patients with contraindications for or no benefit from traditional systemic drugs [53]. Real-life studies highlight the clinical benefits of these drugs in different populations [54] and provide evidence that even a change from IL−17 inhibitors to TNF-α or IL−12/23 inhibitors can be a safe and effective therapeutic strategy for long lasting psoriasis [55]. Notably, even in our cohort of psoriatic patients with AKI, representing a group of patients with severe comorbidities, we detected an increase in the use of biologics over the observed period of time, reflecting the more broadly and successful use over the years also in multi-morbid patients.

The strengths of this study include the high number of AKI patients, who were coded during the observational period, providing a representative group of AKI patients with concomitant psoriasis. Furthermore, we focused on hard endpoints such as in-hospital death and in-hospital adverse events, which are very unlikely to be miscoded or not coded. Nevertheless, there are major limitations regarding our study that require consideration: First, the study results are based on ICD and OPS discharge codes of hospitalized patients, which might lead to an under-reporting or under-coding. Co-medications are not coded in the database and therefore possible drug-related side effects could be part of our study. Second, we can only provide data from the time of hospitalization and have no data about later follow-ups. Third, in only 0.3% AKI cases, psoriasis was coded additionally, which lies far under the known incidence for psoriasis in industrialized nations (2–3%) [2]. However, it is not expected that psoriasis has to be equally prevalent in all special populations, confirmed by other studies reporting even smaller prevalence [56]. Fourth, no exact classification of the psoriatic stage is given. Finally, we were able to study the association between variables registered during hospitalization, but had no information on their temporal or causal relationship.

In our cohort, hospitalizations for AKI in patients with psoriasis occurred in median 6 years earlier in life than in those AKI patients without psoriasis. Despite the younger age, the prevalence of CKD was significantly higher in AKI patients with psoriasis and the cardiovascular profile of patients with psoriasis was substantially aggravated. These findings indicate that psoriasis patients are prone to developing chronic and acute kidney failure as well as cardiovascular diseases, at earlier stages in their lifetime. Moreover, we found a significantly higher use of kidney replacement therapy in patients with psoriasis, indicating a more severe course of acute kidney failure without short-time improvement in these patients. It is known, that patients with severe psoriasis lose a span of three to six years of lifetime [57]. Psoriasis is accompanied by increased number of comorbidities including particularly an aggravated cardiovascular profile confirmed by our study data. Therefore, awareness for these links should be enhanced and a close cooperation of dermatologists with specialists of other specialist disciplines such as nephrologists and cardiologist is needed to improve in the management and care of psoriatic patients, keeping in mind that psoriasis affects the body far beyond the skin.

## Figures and Tables

**Table 1:** Baseline characteristics, treatment, and outcome of patients with acute kidney injury (AKI) stratified by concomitant psoriasis (cumulative data of the years 2005–2016).

Parameters	All Patients (*n* = 3,162,449)	AKI with Psoriasis (*n* = 11,985; 0.4%)	AKI without Psoriasis (*n* = 3,150,464; 99.6%)	*p*-Value
Age (years)	76 (67–83)	70 (60–78)	76 (67–83)	<0.001
Sex (female)	1,449,815 (45.8%)	4750 (39.6%)	1,445,065 (45.9%)	<0.001
Obesity	279,170 (8.8%)	2312 (19.3%)	276,858 (8.8%)	<0.001
**Comorbidities**				
Coronary artery disease	813,696 (25.7%)	3204 (26.7%)	810,492 (25.7%)	0.012
Malignancy	541,108 (17.1%)	1463 (12.2%)	539,645 (17.1%)	<0.001
Heart failure	1,158,990 (36.6%)	4508 (37.6%)	1,154,482 (36.6%)	0.028
Chronic obstructive pulmonary disease	404,619 (12.8%)	2175 (18.1%)	402,444 (12.8%)	<0.001
Diabetes mellitus	1,112,335 (35.2%)	5099 (42.5%)	1,107,236 (35.1%)	<0.001
Essential arterial hypertension	1,425,566 (45.1%)	6102 (50.9%)	1,419,464 (45.1%)	<0.001
Atrial fibrillation/-flutter	985,758 (31.2%)	3660 (30.5%)	982,098 (31.2%)	0.136
Deep vein thrombosis or thrombophlebitis	55,618 (1.8%)	274 (2.3%)	55,344 (1.8%)	<0.001
Chronic kidney disease Stage 1	63,514 (2.0%)	267 (2.2%)	63,247 (2.0%)	0.088
Stage 2	139,711 (4.4%)	709 (5.9%)	139,002 (4.4%)	<0.001
Stage 3	573,569 (18.1%)	2380 (19.9%)	571,189 (18.1%)	<0.001
Stage 4	261,616 (8.3%)	971 (8.1%)	260,645 (8.3%)	0.506
Stage 5	109,487 (3.5%)	490 (4.1%)	108,997 (3.5%)	<0.001
**Dialysis and Type of Procedure**				
Dialysis general	430,856 (13.6%)	1956 (16.3%)	428,900 (13.6%)	<0.001
Hemofiltration	100,537 (3.2%)	453 (3.8%)	100,084 (3.2)	<0.001
Hemodialysis	365,604 (11.6%)	1684 (14.1%)	363,920 (11.6%)	<0.001
Hemodiafiltration	122,393 (3.9%)	554 (4.6%)	121,839 (3.9%)	<0.001
**Serious Adverse events through Hospitalization**				
Gastro-intestinal bleeding	123,962 (3.9%)	509 (4.2%)	123,453 (3.9%)	0.065
Intracranial bleeding	17,022 (0.5%)	78 (0.7%)	16,944 (0.5%)	0.088
Myocardial infarction	206,810 (6.5%)	562 (4.7%)	206,248 (6.5%)	<0.001
Ischemic Stroke	70,796 (2.2%)	237 (2.0%)	70,559 (2.2%)	0.057
Pulmonary embolism	51,699 (1.6%)	195 (1.6%)	51,504 (1.6%)	0.995
Transfusion of erythrocytes	973,186 (30.8%)	4175 (34.8%)	969,011 (30.8%)	0.001
**Primary Endpoint**				
In-hospital mortality	913,283 (28.9%)	2645 (22.1%)	910,638 (28.9%)	<0.001

**Table 2:** Impact of psoriasis on different serious adverse events through hospitalization in patient with acute kidney injury (AKI).

	Univariate Regression Model		Multivariate Regression Model *	
	OR (95% CI)	*p*-Value	OR (95% CI)	*p*-Value
In-hospital mortality	0.70 (0.67–0.73)	<0.001	0.75 (0.72–0.79)	<0.001
Acute myocardial infarction	0.70 (0.65–0.77)	<0.001	0.62 (0.57–0.68)	<0.001
Ischemic Stroke	0.88 (0.77–1.02)	0.053	0.85(0.75–0.97)	0.013
Deep venous thrombosis or thrombophlebitis	1.31 (1.16–1.48)	<0.001	1.29 (1.14–1.45)	<0.001
Pulmonary embolism	0.99 (0.86–1.15)	0.947	0.94 (0.81–1.08)	0.354
Intracerebral bleeding	1.21 (0.97–1.510)	0.092	0.97 (0.77–1.22)	0.802
Gastro-intestinal bleeding	1.09 (0.99–1.19)	0.064	1.06 (0.97–1.16)	0.196
Transfusion of blood constituents	1.20 (1.16–1.25)	<0.001	1.07 (1.03–1.20)	0.001
Initiation of dialysis	1.24 (1.18–1.30)	<0.001	0.93 (0.88–0.98)	0.004

* Adjusted for age, sex, cancer, coronary artery disease, chronic obstructive pulmonary disease, essential arterial hypertension, diabetes mellitus, and malignancy.

## Supplementary Materials

The following are available online at https://www.mdpi.com/2077-0383/9/9/3004/s1, Table S1: Baseline characteristics, treatment and outcome of patients with acute kidney injury and psoriasis stratified by survival status (cumulative data of the years 2005–2016), Figure S1: Time trends of sex distribution in AKI patients with concomitant psoriasis, Figure S2: Time trends of comorbidities in patients hospitalized with AKI with concomitant psoriasis.
