# Supplementary material for: Psoriasis and Its Impact on In-Hospital Outcome in Patients Hospitalized with Acute Kidney Injury
Source: J Clin Med. 2020 Sep 17;9(9):3004. doi: 10.3390/jcm9093004 (PMC7563226; doi:10.3390/jcm9093004)
Supplement: Supplementary file 1 [file jcm-09-03004-s001.pdf]

Supplementary Materials

Table S1. Baseline characteristics, treatment and outcome of patients with acute kidney injury and psoriasis stratified by survival status (cumulative data of the years 2005-2016).

Parameters	AKI + Psoriasis (*n* = 11,985)	Non-Survivors (*n* = 2,645; 22.1%)	Survivors (*n* = 9,340; 77.9%)	*p*-value
Age (years)	**70 (60-78)**	**71 (61-78)**	**70 (60-78)**	**<0.001**
Sex (female)	**4750 (39.6%)**	**980 (37.1%)**	**3770 (40.4%)**	**0.002**
Obesity	**2312 (19.3%)**	**424 (16.0%)**	**1888 (20.2%)**	**<0.001**
**Comorbidities**				
**Coronary artery disease**	3204 (26.7%)	716 (27.1%)	2488 (26.6%)	0.655
**Malignancy**	**1463 (12.2%)**	**492 (18.6%)**	**971 (10.4%)**	**<0.001**
**Heart failure**	**4508 (37.6%)**	**1140 (43.1%)**	**3368 (36.1%)**	**<0.001**
COPD	**2175 (18.1%)**	504 (19.1%)	1671 (17.9%)	0.170
Diabetes mellitus	**5099 (42.5%)**	**1062 (40.2%)**	**4073 (43.2%)**	**0.005**
**Essential arterial hypertension**	**6102 (50.9%)**	**1182 (44.7%)**	**4920 (52.7%)**	**<0.001**
**Atrial fibrillation/-flutter**	**3660 (30.5%)**	**930 (35.2%)**	**2730 (29.2%)**	**<0.001**
**Deep vein thrombosis**	**274 (2.3%)**	**49 (1.9%)**	**225 (2.4%)**	**0.107**
CRI 1	267 (2.2%)	53 (2.0%)	214 (2.3%)	0.411
**CRI 2**	**709 (5.9%)**	**129 (4.9%)**	**580 (6.2%)**	**0.011**
**CRI 3**	**2380 (19.9%)**	**384 (14.5%)**	**1996 (21.4%)**	**<0.001**
CRI 4	971 (8.1%)	210 (7.9%)	761 (8.1%)	0.745
CRI 5	490 (4.1%)	109 (4.1%)	381 (4.1%)	0.915
**Dialysis and type of procedure**				
**Dialysis general**	**1956 (16.3%)**	**759 (28.7%)**	**1197 (12.8%)**	**<0.001**
**Haemofiltration**	**453 (3.8%)**	**240 (9.1%)**	**213 (2.3%)**	**<0.001**
**Haemodialyse**	**1684 (14.1%)**	**606 (22.9%)**	**1078 (11.5%)**	**<0.001**
**Haemodiafiltration**	**554 (4.6%)**	**292 (52.7%)**	**262 (2.8%)**	**<0.001**
**Serious adverse events through hospitalization**				
**Gastro-intestinal bleeding**	**509 (4.2%)**	**168 (6.4%)**	**341 (3.7%)**	**<0.001**
**Intracranial bleeding**	**78 (0.7%)**	**35 (1.3%)**	**43 (0.5%)**	**<0.001**
**Myocardial infarction**	**562 (4.7%)**	**196 (7.4%)**	**366 (3.9%)**	**<0.001**
**Stroke**	**237 (2.0%)**	**80 (3.0%)**	**157 (1.7%)**	**<0.001**
**Pulmonary embolism**	**195 (1.6%)**	**71 (2.7%)**	**124 (1.3%)**	**<0.001**
**Transfusion of erythrocytes**	**4175 (34.8%)**	**1432 (54.1%)**	**2743 (29.4%)**	**<0.001**

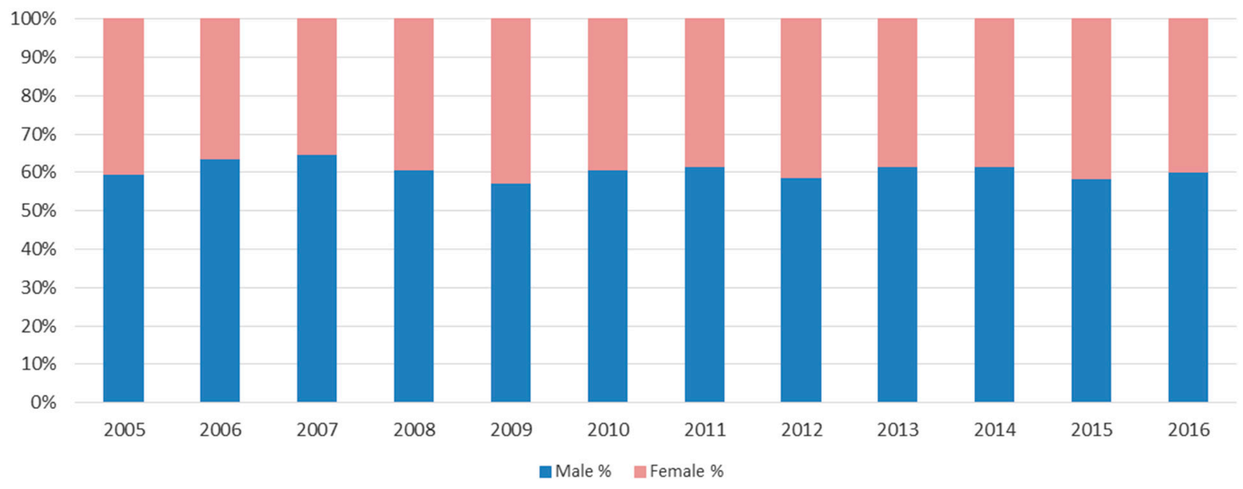

Figure S1. Time trends of sex distribution in AKI patients with concomitant psoriasis.

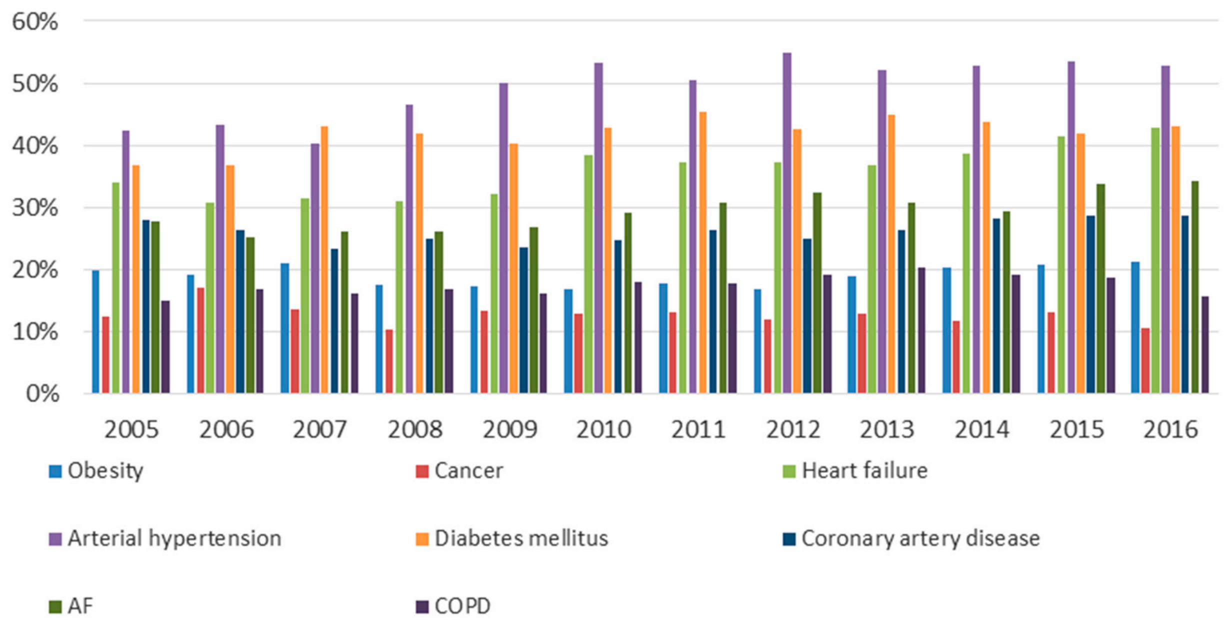

Figure S2. Time trends of comorbidities in patients hospitalized with AKI with concomitant psoriasis.
